# Comparative Transcriptome Analysis of Deep-Rooting and Shallow-Rooting Potato (*Solanum tuberosum* L.) Genotypes under Drought Stress

**DOI:** 10.3390/plants11152024

**Published:** 2022-08-03

**Authors:** Tianyuan Qin, Chao Sun, Ali Kazim, Song Cui, Yihao Wang, Dormatey Richard, Panfeng Yao, Zhenzhen Bi, Yuhui Liu, Jiangping Bai

**Affiliations:** 1 Gansu Provincial Key Laboratory of Aridland Crop Science, College of Agronomy, Gansu Agricultural University, Lanzhou 730070, China; qty1637835362@163.com (T.Q.); sunc@gsau.edu.cn (C.S.); xiaohao9639@163.com (Y.W.); rmddormatey@gmail.com (D.R.); yaopf@gsau.edu.cn (P.Y.); bizhen925@sina.com (Z.B.); lyh88311@sohu.com (Y.L.); 2 National Institute for Genomics and Advanced Biotechnology, National Agricultural Research Centre, Park Road, Islamabad 45500, Pakistan; kazim76@gmail.com; 3 School of Agriculture, Middle Tennessee State University, Murfreesboro, TN 37132, USA; song.cui@mtsu.edu

**Keywords:** potato, drought stress, transcriptome, MAPK signaling pathway, plant hormone signaling pathways

## Abstract

The selection and breeding of deep rooting and drought-tolerant varieties has become a promising approach for improving the yield and adaptability of potato (*Solanum tuberosum* L.) in arid and semiarid areas. Therefore, the discovery of root-development-related genes and drought tolerance signaling pathways in potato is important. In this study, we used deep-rooting (C119) and shallow-rooting (C16) potato genotypes, with different levels of drought tolerance, to achieve this objective. Both genotypes were treated with 150 mM mannitol for 0 h (T0), 2 h (T2), 6 h (T6), 12 h (T12), and 24 h (T24), and their root tissues were subjected to comparative transcriptome analysis. A total of 531, 1571, 1247, and 3540 differentially expressed genes (DEGs) in C16 and 1531, 1108, 674, and 4850 DEGs in C119 were identified in T2 vs. T0, T6 vs. T2, T12 vs. T6, and T24 vs. T12 comparisons, respectively. Gene expression analysis indicated that a delay in the onset of drought-induced transcriptional changes in C16 compared with C119. Functional enrichment analysis revealed genotype-specific biological processes involved in drought stress tolerance. The metabolic pathways of plant hormone transduction and MAPK signaling were heavily involved in the resistance of C16 and C119 to drought, while abscisic acid (ABA), ethylene, and salicylic acid signal transduction pathways likely played more important roles in C119 stress responses. Furthermore, genes involved in root cell elongation and division showed differential expression between the two genotypes under drought stress. Overall, this study provides important information for the marker-assisted selection and breeding of drought-tolerant potato genotypes.

## 1. Introduction

Potato (*Solanum tuberosum* L.) is one of the four major food crops in the world and is commonly grown as a staple food crop in arid and semiarid regions with an annual average precipitation of less than 500 mm [1]. In these areas, the yield and quality of potato tubers are limited by many biotic and abiotic stresses, among which long-term or seasonal drought stress have detrimental effects on canopy growth as well as tuber yield and market value [2,3]. Therefore, one of the major goals of breeding programs conducted in arid/semiarid regions is the development of strategies and techniques that can improve the drought tolerance of potato. Investigation of the mechanisms of root growth and development in different genotypes under drought stress using advanced genomic approaches has become an urgent task and research hotspot in the past decade, and could provide important insights, which could facilitate the marker-assisted selection of drought-tolerant potato cultivars [4]. Furthermore, there is a pressing need for a systematic comparative transcriptome analysis of potato cultivars with varying drought tolerance levels using different analytical methods, with a focus on molecular signaling/functioning pathway analyses.

In general, potato shows higher water use efficiency (WUE) than other C3 plants [5]; however, it has a shallow root system and shows high sensitivity towards osmotic stress, and therefore is more vulnerable to severe soil moisture depletion [6]. Therefore, potato is commonly considered as a drought-sensitive crop. Attempts have been made to increase the drought tolerance of potato by increasing root production, reducing transpiration, and improving the plant WUE and fertilizer use efficiency [7]. A number of studies mainly focused on the responses of different potato root phenotypes to drought stress and investigated the stress-resistant physiological and biochemical indices of potato [8,9]. However, there are few investigations about the extent of variation in the drought stress response among genotypes with different root architecture, and wide-ranging systematic molecular approaches have not been fully used for the exploration of drought stress-responsive genes in potato [10,11]. 

With the completion of the reference genome sequence of doubled-haploid potato [12], next-generation sequencing approaches such as transcriptomics, proteomics, and metabolomics, in conjunction with data analytics, could now be used to study the mechanisms of abiotic and biotic stress resistance in potato [13,14,15,16]. Moreover, full-length RNA sequencing can be directly performed without sequence splicing, which greatly reduces the systematic errors caused by in vitro reverse transcription. In addition, as the cost of transcriptome sequencing declines, sequencing and comprehensive analysis of multi-samples from multiple condition combinations become possible. These scientific and technological advancements provide excellent opportunities for investigating the genome-wide variation in nucleotide sequences and gene expression levels governing the physiological traits of different potato cultivars [17,18]. However, further research effort is warranted for investigating the effects of genotype × environment interactions on potato cultivars, particularly in the semiarid environment. 

In this study, seedings of two potato genotypes with different root configurations and drought resistance levels were exposed to drought stress for 0 h (T0), 2 h (T2), 6 h (T6), 12 h (T12), and 24 h (T24), and then phenotyped. Additionally, various biochemical indices were determined, and the roots of both genotypes were subjected to transcriptome sequencing and systematic analyses to answer two main research questions in depth: (1) what are the major transcriptional-response-related differences between the two genotypes in response to different durations of drought stress; (2) what is the genetic basis of the differences between the ability of these two genotypes to cope with drought stress? Based on bioinformatics analysis, the results of this study are expected to provide great insight into the molecular mechanism controlling the response of potato plants to drought stress and into the relationship between root configuration and drought resistance, which will eventually help to explore new molecular techniques for breeding drought-resistant cultivars.

## 2. Results

### 2.1. Differences in Physiological and Biochemical Indexes between the Two Genotypes

**Figure 1:**
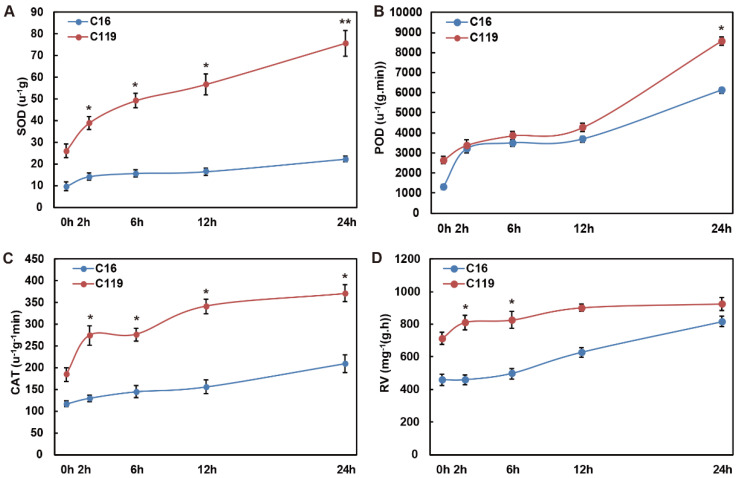
Measurements of physiological and biochemical indices in C16 and C119 roots under drought stress. (**A**–**D**) SOD (**A**), POD (**B**), CAT (**C**), and RV (**D**) activities in C16 (blue line) and C119 (red line) at T0, T2, T6, T12, and T24. Asterisks indicate significant differences between the two genotypes at the same time point (* *p* < 0.05, ** *p* < 0.01; LSD test).

To study the effects of different drought stress conditions on tube seedlings of the two potato genotypes (C16 and C119), we quantified the physiological and biochemical indices related to stress resistance, including superoxide dismutase (SOD), peroxidase (POD), and catalase (CAT) activities and root activity (RV) (Figure 1). SOD, POD, CAT, and RV are usually involved in alleviating the damage caused by external stress [19]. With the increase in the duration of drought stress, the SOD activity level increased slightly in C16 at T2, remaining at a low level thereafter, but increased rapidly in C119 (Figure 1A). Moreover, the activity of SOD was significantly higher in C119 than in C16 at all time points (*p* < 0.05), and the difference between the two genotypes became the most pronounced at T24 (*p* < 0.01) (Figure 1A). The POD activity level increased in C16 until T2, and then remained stable until T12, before rising again gradually until T24. In C119, the POD activity level showed no considerable change until T12, and then increased rapidly to levels significantly higher than those in C16 at T24 (*p* < 0.05) (Figure 1B). The activity of CAT in C16 plants increased gradually throughout the drought stress treatment in C16 (Figure 1C). By contrast, in C119 plants, CAT activity increased significantly until T2, and then remained significantly higher than that in C16 (*p* < 0.05) throughout the entire duration experiment to cope with drought stress (Figure 1C). The level of RV increased gradually in C16 but rapidly in C119 plants after the start of drought stress, resulting in significantly higher levels in C119 than in C16 at T2 and T6 (*p* < 0.05) (Figure 1D). Evidently, despite the consistent increase in the selected biochemical indicators with the increase in treatment time, the drought stress response patterns of the two genotypes varied greatly. In general, C119 responded more rapidly to drought stress than C16, and the activities of protective enzymes were consistently higher in C119 than in C16 across the different treatment times. These results suggest that C119 is more drought tolerant than C16.

### 2.2. Validation of RNA-seq Data

**Figure 2:**
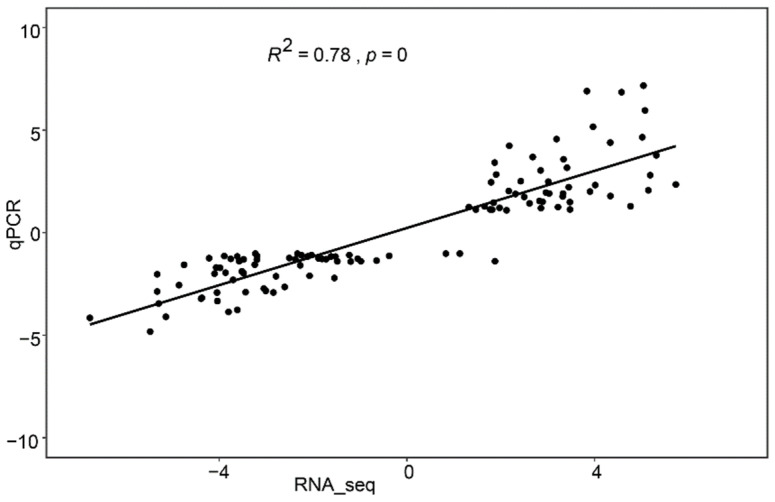
Correlation between the expression levels of 12 randomly selected genes determined by qPCR and those determined by RNA-seq. The correlation between RNA-seq (*x*-axis) and qPCR (*y*-axis) data was analyzed by the Pearson test (R^2^ = 0.78, *p* < 0.01).

To evaluate the reliability of the RNA-seq data, 12 genes with different expression patterns were randomly selected, and their expression levels were examined by real-time quantitative RT- PCR (qPCR). The results showed a strong correlation between the RNA-seq and qPCR data (R^2^ = 0.78, *p* = 0, Figure 2). The transcript levels of all 12 genes, accounting for 78.5% of the analyzed genes, including *ABRE* binding factor and Auxin response factor, were in agreement with the RNA-seq data (Figure S1). These results demonstrate that our RNA-seq data are highly reliable.

### 2.3. Expression Pattern and Functional Enrichment Analyses of Drought-Responsive Genes 

**Figure 3:**
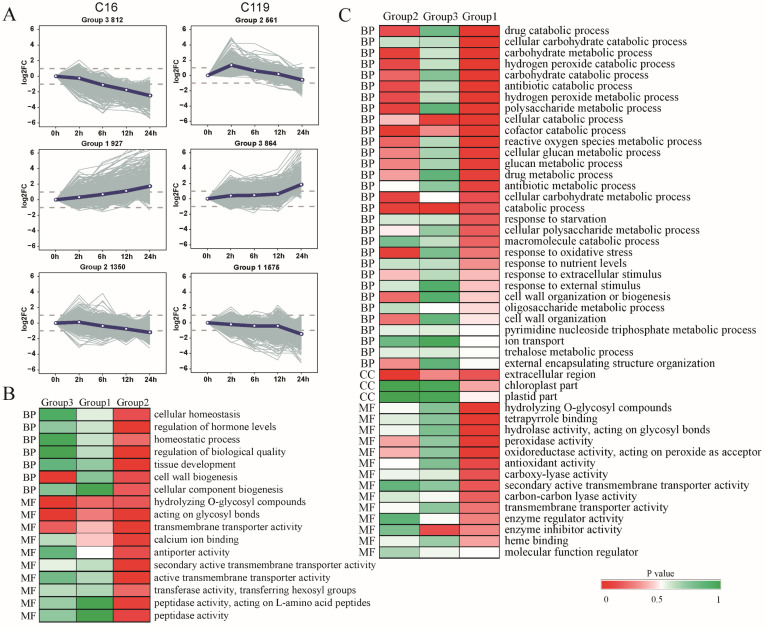
Gene expression patterns and GO enrichment analyses of genes clustered into three main groups in C16 and C119. (**A**) Patterns of gene expression across five time points in C16 and C119, as inferred by STEM analysis. In each frame, light gray lines represent the expression pattern of each gene, while the blue line represents the overall expression trend of all genes. The number of genes showing each pattern is indicated above the frame. (**B**,**C**) GO enrichment analysis of genes in three significant clusters in C16 (**B**) and C119 (**C**). The p-value indicates the significance of the most represented GO-slims in each main cluster. Red color indicates significantly upregulated genes and green color indicates significantly downregulated genes.

Using comparative transcriptome analysis of root tissues, we identified 531, 1571, 1247, and 3540 differentially expressed genes (DEGs) in C16 and 1531, 1108, 674, and 4850 DEGs in C119 in T2 vs. T0, T6 vs. T2, T12 vs. T6, and T24 vs. T12 comparisons, respectively. Using the STEM software, DEGs identified in C16 and C119 at different time points were clustered into six groups (Groups 1–6), based on their expression profiles. The gene expression profiles displayed considerable differences between the two genotypes in response to the duration of drought stress (Figure 3A). In C119, the DEGs were significantly overrepresented at T2 (Groups 1, 2, and 3; *p* < 0.05), whereas a significant increase in major transcriptional changes occurred at T24 in C16 (Groups 1, 2, and 3; *p* < 0.05). These results suggest that the transcriptional response to drought stress was delayed in C16 compared with C119, and the DEGs detected at T2 potentially play an important role in the drought resistance of C119.

Gene Ontology (GO)- and Kyoto Encyclopedia of Genes and Genomes (KEGG)-based classifications revealed several overexpressed gene groups. In C119, genes involved in cellular glucose metabolism, catabolism, oxidative stress, and extracellular component transmutation were enriched in Group 2 (Figure 3A,C). Among these genes, those showing increased expression levels at T2 and decreased expression levels at T6, T12, and T24 were more sensitive in the early stages of drought stress. A similar pattern was previously observed in Arabidopsis [20], where many genes responsive to stress and stimuli were induced within 2 h after exposure to drought stress rather than within weeks. In C119, GO terms such as cellular catabolic processes and enzyme inhibitor activities were enriched among Group 3 genes, and GO terms including cofactor metabolism, reactive oxygen metabolism, cellular glucose metabolism, antibiotic metabolism, hydrolysis of O-glycosyl hydrolase activity, tetrapyrrole binding, hydrolase activity, action Glycosyl bond, peroxidase activity, oxidoreductase activity, peroxide as receptor, and antioxidant activity were enriched among Group 1 genes (Figure 3A,C). The expression levels of these genes Group 3 began to rise after T0, remained high at T2 and T6, and then increased rapidly after T12, eventually reaching a peak at T24, while those of Group 1 genes began to decline slowly after T0, remained at a low level from T2 to T12, and then declined rapidly, reaching the lowest level at T24 (Figure 3A,C). However, in C16, GO terms such as cell wall biosynthesis, hydrolase activity of the O-glycosyl compound, hydrolase activity, glycosyl bond, and transmembrane transporter activity were enriched among Group 3 genes, while GO terms such as regulating cell homeostasis, hormone levels, biomass regulation, tissue development, cell wall biosynthesis, cell component biogenesis, cross membrane transporter activity, secondary active transmembrane transporter activity, transmembrane transport activity, peptidase activity, L-amino acid peptides, and peptidase activity were enriched among Group 2 genes (Figure 3A,B). These results revealed considerable differences in drought-responsive genes and pathways between the two genotypes. The differences in gene expression patterns between the two genotypes correspond to their different subcellular distributions and chemical forms after drought stress treatment.

### 2.4. Deep-Rooting Genotypes Respond Faster to Drought Stress than Shallow-Rooting Genotypes 

**Figure 4:**
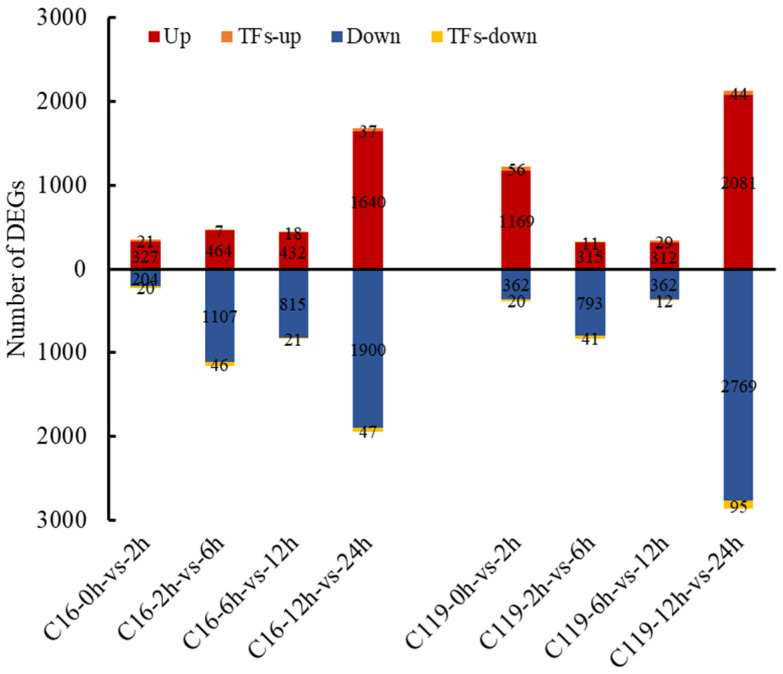
Pairwise comparison of DEGs among the five drought treatment time points in C16 and C119. Red column represents upregulated genes; blue column represents downregulated genes; orange column represents upregulated transcription-factor-encoding genes; and yellow column represents downregulated transcription-factor-encoding genes.

Pairwise comparisons of DEGs among the five drought treatment time points revealed considerable differences between C16 and C119 (Figure 4). After removing duplicate genes, a total of 1286 and 1498 DEGs across all time points were assigned to 239 and 305 GO terms, respectively, in C16 and C119 (Figure S2). GO enrichment analysis also indicated significant differences in the number of genes enriched in the two genotypes under different GO terms (Figure S2).

Compared with T0, the T2 time point showed only 327 upregulated and 204 downregulated genes in C16, and 1169 upregulated and 362 downregulated genes in C119 (Figure 4). In addition, compared with C16, more genes were significantly enriched in all three GO categories (biological process, cellular component, and molecular function), particularly in terms of stress tolerance, in C119 of T2 (Figure S2A). GO terms including oxidative stress response, ion transport, and response to external stimuli were enriched in the biological process category, and only one GO term (metabolic process) was significantly enriched at T2 in C119 (*p* < 0.05) (Figure S2A). Accordingly, compared with C16, more gene classes responsive to antioxidant stress were excessively enriched in C119 (Figure 3C). These results imply a trade-off between cell wall thickening or regionalization and anti-oxidation in C119 to cope with the damage caused by drought stress. These results also indicate that changes in the transcript levels of drought-responsive genes in C119 were more active during the initial phase of the drought treatment, which is consistent with the performance of Group 2 genes (Figure S2A and Figure 3A).

In the T6 vs. T2 comparison, 464 and 315 upregulated genes and 1107 and 793 downregulated genes were identified in C16 and C119, respectively (Figure 4). Compared with expressed levels at T2, the genes induced by drought stress in C119 were downregulated at T6 (Figure S2B). In Arabidopsis, the expression levels of early stress-responsive genes were also downregulated at subsequent time points [19,20] indicating that the genetic responses of plants to drought stress depend greatly on the severity and duration of the stress. In C16, a class of genes associated with extracellular region were significantly induced at T6 compared with T2 (Figure S2B). The slower activation of early stress-responsive genes in C16 compared with C119 was thought to be caused by the delayed perception of stress signals by C16 roots. 

In the T12 vs. T6 comparison, 432 and 312 upregulated genes and 815 and 362 downregulated genes were identified in C16 and C119, respectively (Figure 4). Compared with gene expression levels at T6, the drought stress-responsive genes were downregulated at T12 in C119 but significantly upregulated in C16 (Figure S2C). At T12, more genes are enriched in the GO terms of plastids, membrane protein complexes, and thylakoids in C16 than in C119, indicating that the drought stress responses were stronger and more significant in C16 than in C119 at T12 (*p* < 0.05) (Figure S2C). 

In the T24 vs. T12 comparison, 1640 and 2081 upregulated genes and 1900 and 2769 downregulated genes were identified in C16 and C119, respectively (Figure 4). In C119, the drought stress-responsive genes were slightly upregulated at T24 compared with T12 (Figure S2D). At T24, the responses to drought stress were more significantly pronounced in C119 compared with C16 (*p* < 0.05, Figure S2D), particularly with respect to genes involved in tetrapyrrole binding, transmembrane transporter activity, and oxidative stress.

Overall, C119 showed a rapid response to drought stress at the transcriptional level of antioxidant-related pathways and reached a higher level in the early stage of stress, whereas C16 exhibited a relatively delayed response. The key genes involved in these pathways could be investigated further in the future. In addition, C16 showed a significant enrichment of cell components at the middle stage of the treatment, although its biological function is largely unknown and therefore should be explored further.

### 2.5. KEGG Enrichment Analysis of DEGs

In an organism, each biological process and its regulatory pathway are usually controlled by multiple genes and their interactions. KEGG enrichment analysis is a commonly used method for investigating the functions and expression patterns of genes influenced by certain abiotic or biotic factors, and the results are usually shown in the form of scatter plots [21]. Additionally, the rich factor, q-values, and number of enriched genes are used to measure the degree of KEGG enrichment. Rich factor is the ratio of the number of DEGs to the number of all annotated genes in a particular pathway entry [22]. The q-value represents the p-value after correction for multiple hypothesis testing, and ranges from 0 to 1 [21]. The closer the q-value to zero, the more significant the enrichment of DEGs.

**Figure 5:**
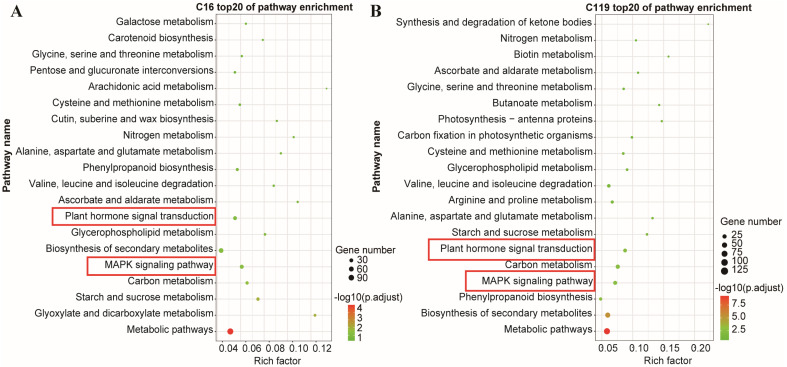
Top 20 KEGG pathways. (**A**) C16; (**B**) C119. The x-axis shows the rich factor; the *y*-axis shows the KEGG pathways.

DEGs identified in the two genotypes at all drought time points were analyzed using the KEGG enrichment analysis. Many processes were significantly enriched in both genotypes, and the top 20 most enriched pathways are shown in Figure 5. Among them, the genes involved in mitogen-activated protein kinase (MAPK) and plant hormone transduction pathways were closely related to root growth and development as well as stress resistance, which is similar to the findings reported in previous studies on model plants and Gramineae species [23]. Although the MAPK and plant hormone transduction pathways were enriched in both genotypes, the expression patterns of genes involved in these pathways showed significant differences between the two genotypes (Figure 5A,B). Thus, these two KEGG pathways were also considered to be the most influential in controlling the physiological and genetic responses of potato to drought stress and are discussed further in Section 2.6.

### 2.6. Comparison of MAPK Signaling Pathway Related Gene Expression between Shallow and Deep-Rooting Potato Genotypes

**Figure 6:**
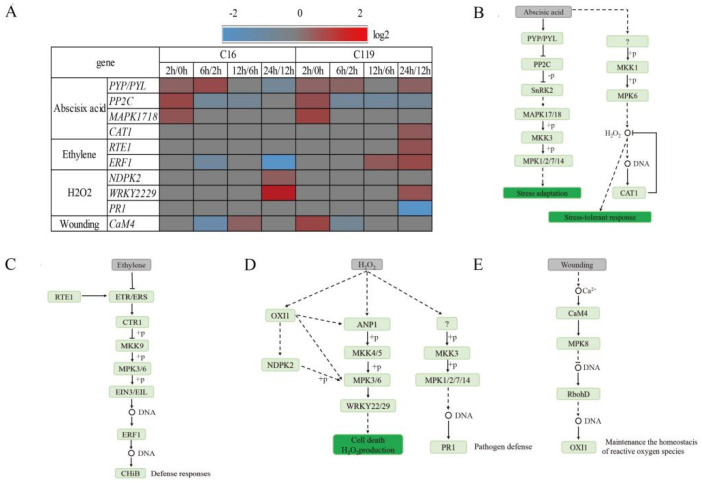
Temporal changes in the expression patterns of genes involved in ABA, ethylene, H_2_O_2_, and wounding signal transduction pathways in response to drought stress. (**A**) Heat map showing the relative transcript levels of genes involved in ABA, ethylene, H_2_O, and wounding signaling under drought treatments. Genes showing greater than 2-fold upregulation or downregulation after drought stress are marked in red and blue, respectively. Unidentified steps are marked in gray. (**B**–**E**) Signal transduction pathways of ABA (**B**), ethylene (**C**), H_2_O_2_ (**D**), and wounding (**E**) in potato.

We found that four *MAPKs* involved in the signal transduction of abscisic acid (ABA), ethylene, hydrogen peroxide (H_2_O_2_), and wounding also responded to the drought treatment, and the response patterns of these genes differed between the two genotypes. Among the ABA–MAPK signal transduction pathway genes, four genes changed expression levels under drought stress; most of these genes showed very similar expression patterns at T2, T6, and T12 between the two genotypes but began to show different expression patterns after T12 (Figure 6A). After T12, the genes encoding PYP/PYL receptor proteins were downregulated in C16 by were upregulated in C119. In C119, ABA can also induce another MAPK cascade reaction, namely MKK1–MPK6, which can induce the accumulation of H_2_O_2_, thereby activating a series of stress-response pathways (Figure 6A,B). 

Change in ethylene content in the MAPK signaling pathway activates the MKK9–MPK3/6 cascade reaction, as well as downstream genes such as those encoding ethylene-responsive factors (*ERFs*). The expression of *ERF1* was downregulated in C16 at T6 and T24, thereby slowing its defense response to stress. In C119, the expression of *ERF1* was still significantly upregulated at T12 and T24. In addition, we also found that the expression level of *RTE1*, which controls the epigenetic regulation of *ERF1*, was significantly upregulated at T24 (Figure 6A,C). 

According to our results, H_2_O_2_ regulates the MAPK signaling cascade ANP1–MKK4/5–MPK3/6 through the kinase OXI1. Genes encoding the NDPK2 protein and WRKY22/29 transcription factors were upregulated in C16 at T24. In addition, the MKK3–MPK1/2/7/14 cascade reaction was also activated in C119, and the *PR1* gene was involved in the anti-adverse reaction (Figure 6A,D). 

The *CaM4* gene was upregulated in C119 at T2. In C16, however, the expression of *CaM4* was slightly increased until T12, and its degree of response to drought stress was delayed compared with C119 (Figure 6A,E). 

### 2.7. Plant Hormone Signaling Pathways Exhibit Differential Responses to Drought Stress between Shallow and Deep-Rooting Genotypes 

**Figure 7:**
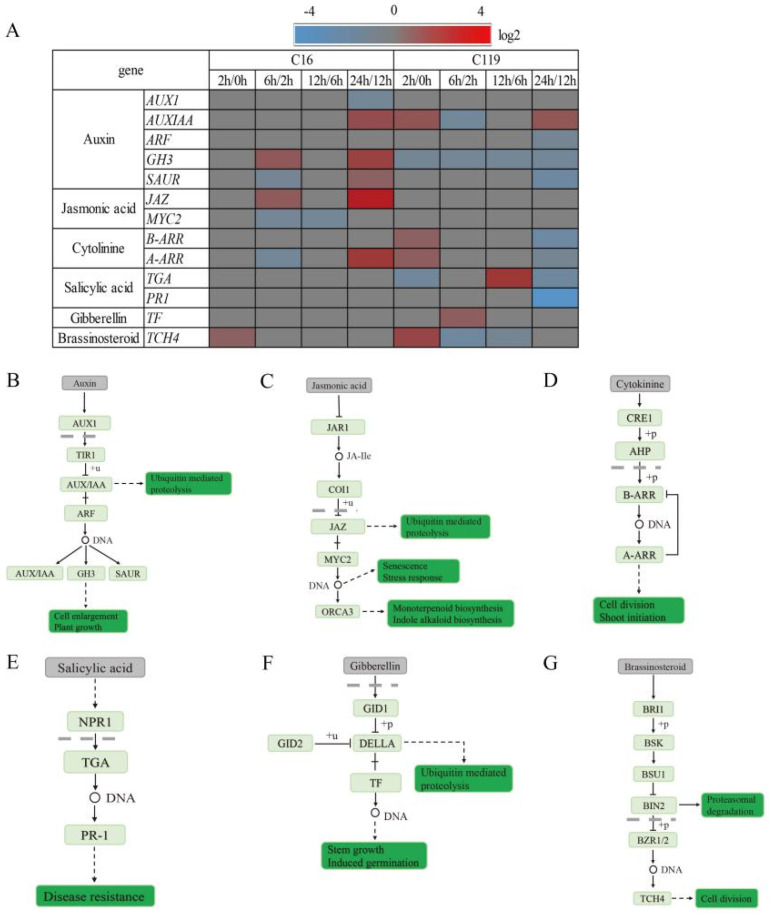
Changes in the expression of genes involved in auxin, JA, CK, SA, GA, and brassinolide signaling pathways in response to drought stress. (**A**) Heat map showing the relative transcript levels of genes involved in auxin, JA, CK, SA, GA, and brassinolide signaling pathways in potato plants treated with drought stress. Genes showing >2-fold upregulation or downregulation after drought stress are marked in red and blue, respectively. Unidentified steps are marked in gray. (**B**–G) Signal transduction pathways of auxin (**B**), JA (**C**), CK (**D**), SA (**E**), GA (**F**), and brassinolide (**G**) in potato.

We found that the signaling pathways of six plant hormones, including auxin, jasmonic acid (JA), cytokinin (CK), salicylic acid (SA), gibberellin (GA), and brassinolide, were involved in the drought stress response, although the response patterns differed between the two genotypes. In C16, the expression level of the gene encoding GH3 was slightly upregulated at T6 and T24, while that of *AUX1*, which encodes *AUX*/*IAA*, was downregulated at T24. However, in C119, the expression level of *AUX*/*IAA* was upregulated at T2 and T24, leading to enhanced cell expansion and growth at the early (T2) and late (T24) stages of drought stress (Figure 7A,B).

The expression of *JAZ* was upregulated in C16 at T6 and T24, which resulted in the expression of downstream JA-responsive genes and consequently the regulation of plant senescence and drought stress response. In C119, however, we did not find any significant changes in the expression levels of related genes (Figure 7A,C).

CK plays an important role in plant resistance to drought stress. In C16, type-A Arabidopsis Response Regulators (*A-ARRs*) were downregulated at T6 but upregulated at T24. An opposite trend was observed in C119, where type-B ARRs (*B-ARRs*) and *A-ARRs* were upregulated at T2 but downregulated at T24 (Figure 7A,D).

The SA signal transduction pathway and its effect on *TGA* transcription factors has been well documented previously. In C119, the *TGA* gene was downregulated at T2, which likely compromised plant stress resistance, and then was significantly upregulated at T12. Similarly, the expression levels of *TGA* and *PR-1* decreased at T24, compromising plant stress resistance. However, in C16, we did not find any change (Figure 7A,E). 

In the GA signal transduction pathway, GA binds to the receptor GID1 in the nucleus, which then binds to the repressor protein DELLA, forming a stable complex. In C119, the expression level of *TF* was upregulated at T6, thereby promoting plant growth. However, in C16, the expression levels of GA signaling genes showed no changes in expression levels (Figure 7A,F). 

In the brassinolide signal transduction pathway, brassinosteroid (BR) binds to the extracellular domain of BRI1, leading to its phosphorylation. At T2, the *TCH4* gene was upregulated in both C16 and C119; however, its expression level changed more in C119 than in C16, which suggests that C119 has an advantage over C16 in maintaining cell elongation and growth at the early stage of drought stress (Figure 7A,G).

### 2.8. Subcellular Localization of StJAZ and StTF Proteins

**Figure 8:**
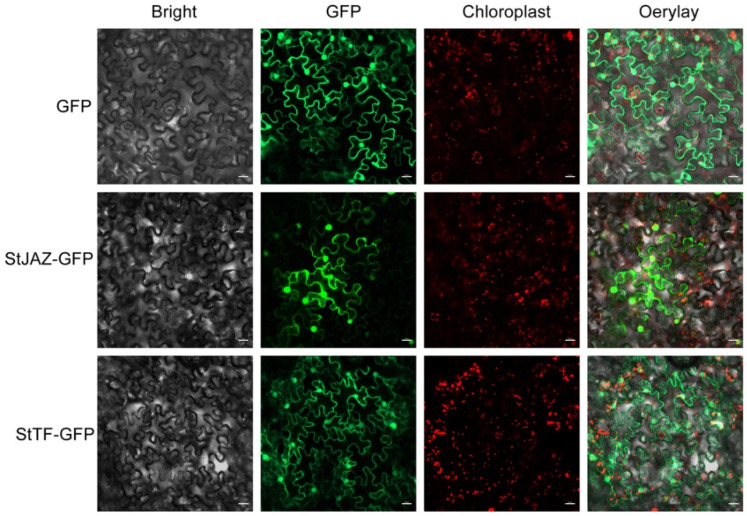
Subcellular localization of *StJAZ* and *StTF* proteins in tobacco leaves. Confocal laser scanning microscopy analysis was undertaken on tobacco epidermal cells transfected with *GFP*-fused *StJAZ* and *StTF* sequences. Transfection with *GFP* alone serves as a negative control. Scale bar, 20 μm.

The *StJAZ* gene was specifically expressed in C16, while the *StTF* gene was specifically expressed in C119 (Figure 7A). Since the physiological function of the protein is extensively associated with its subcellular localization, we determined the localization of *StJAZ* and *StTF* proteins. The nucleotide sequences of *StJAZ* and *StTF* were separately fused to the green fluorescent protein (*GFP*) gene. The fusion construct was expressed in tobacco (*Nicotiana tabacum* L.) leaves, and *GFP* signals were detected by laser confocal scanning microscopy. *StJAZ* and *StTF* localized to the cell membrane and nucleus concurrently (Figure 8). 

## 3. Discussion

Potato crop is subject to a variety of biotic and abiotic stresses during growth and development. Drought stress is one of the major abiotic stresses affecting potato at the germination and seedling stages. Screening for drought-tolerant varieties and research into the drought tolerance mechanisms have significance for potato production. In this study, RNA-seq was used to identify the root genes of the drought-tolerant potato C119 and drought-sensitive potato C16 under drought stress.

### 3.1. Changes in Growth Parameters and Physiological Indicators

The growth parameters and physiological traits of C16 and C119 indicated that both cultivars performed differently under drought stress conditions.

SOD, POD, and CAT are important antioxidant enzymes in plants that eliminate ROS and peroxides induced by stress, inhibit the peroxidation of the plasma membrane, and protect cells from damage [24]. The activity of these enzymes increased in both potato cultivars under stress conditions, and their activity was higher in C119 than in C16 both under control and stress conditions. The increase in SOD, POD, and CAT activities in C119 was greater than that in C16 (Figure 1A–C). Studies have shown that the increase in the activity of these enzymes under stress conditions protects plant cells from oxidative damage emanating from the ROS generated under such conditions [25]. Thus, we conclude that C119 can effectively eliminate free radicals under simulated drought stress.

Root vigor (RV) increased significantly in the roots of both potato cultivars under stress conditions. RV was generally higher in C119 roots than in C16 both under control and stress conditions (Figure 1D). In a previous study, RV was significantly higher in the tolerant potato genotype than in the sensitive genotype under control and drought conditions [25]. It is thus speculated that the higher RV of C119 allows it to maintain more effective physiological and biochemical processes under drought stress.

Based on the results of the physiological assessment of C16 and C119, it can be inferred that stress tolerance mechanisms are activated in potato roots under simulated drought stress. The higher RV under control and stress conditions and the greater increase in SOD, POD, and CAT activities under stress conditions conferred C119 with stronger drought resistance compared with C16. The increase in root vigor, the ability to maintain plasma membrane balance, and the effectiveness in eradicating ROS made C119 more resistant to drought.

### 3.2. MAPK Signaling Pathway in C119 and C16 Response to Drought Stress

The MAPK cascade plays a very important role in intracellular pathogen immunity and abiotic stress signal transduction. The MAPK cascade plays a key role in enzyme activation and inactivation through its phosphorylation and dephosphorylation, respectively; thus, allowing the rapid and specific signal transduction and amplification of external stimuli [26]. We found that four *MAPKs* involved in the signal transduction of ABA, ethylene, H_2_O_2_, and wounding also responded to the drought treatment, and the expression patterns of the corresponding genes differed between the two genotypes. Among the ABA–MAPK signal transduction pathway genes, four genes changed expression levels under drought stress; most of these genes showed very similar expression patterns at T2, T6, and T12 between the two genotypes but began to show different expression patterns after T12 (Figure 6A). After T12, in C16, the genes encoding PYP/PYL receptor proteins were downregulated, preventing the receptor from binding to ABA, and thus preventing the activation of downstream stress adaptation pathways. In C119, however, the expression level of genes encoding PYP/PYL receptor proteins continued to increase; thus, allowing PYP/PYL proteins to bind to the negative regulator PP2C to inhibit its protein phosphatase activity. Our findings are in agreement with those of Krzywińska et al. [27], who showed that PP2C dephosphorylates and inhibits kinase activity of SnRK2. Inhibition of the kinase activity of SnRK2 by PP2C activates the MAPKKK17/18–MKK3–MPK1/2/7/14 cascade [25], which in turn activates the downstream stress-response pathway. In C119, ABA also induced another MAPK cascade, namely MKK1–MPK6, which induced the accumulation of H_2_O_2_, thereby activating a series of stress-response pathways (Figure 6A,B). The regulation of PYP/PYL activity by ABA has been reported in many previous studies [27,28], which are largely in agreement with our findings. Additionally, our findings also indicated that with the increase in the duration of drought stress, the expression levels of genes encoding the same protein and the activation of downstream stress pathways varied greatly between the two genotypes. This suggests that, in addition to the common stress pathway, there exist unique genotype-specific stress pathways that help plants cope with drought stress. Change in ethylene content in the MAPK signaling pathway activates the MKK9–MPK3/6 cascade, which further activates downstream genes, such as *ERFs*, to promote plant defense responses. These results verify that ethylene can enhance plant tolerance by regulating the expression of *ERF1*, as described in a classical study [29]. The expression of *ERF1* was downregulated in C16 at T6 and T24, thereby slowing its defense response to stress. In C119, the *ERF1* gene was significantly upregulated at T12 and T24. In addition, we also found that the expression level of *RTE1*, which controls the epigenetic regulation of *ERF1*, was significantly upregulated at T24, presumably enhancing plant tolerance to ethylene [29] and therefore the resistance to drought stress (Figure 6A,C). Consistent with the above-described findings, C119 showed stronger drought tolerance than C16, although this genotype-level difference has rarely been reported previously.

According to our results, H_2_O_2_ regulates the ANP1–MKK4/5–MPK3/6 signaling cascade through the OXI1 kinase. OXI1 uses the NDPK2 kinase to phosphorylate MPK3/6, which in turn activates the downstream transcription factors WRKY22/29. This is quantitatively in agreement with previous studies [30]. Genes encoding the NDPK2 protein and WRKY22/29 transcription factors were upregulated in C16 at T24, thereby accelerating cell death and H_2_O_2_ accumulation. In addition, the MKK3–MPK1/2/7/14 cascade reaction was shown to be activated, and the *PR1* gene was also involved in the anti-adverse reaction (Figure 6A,D). These results suggest that C119 can activate more cascade reactions than C16 in response to drought stress.

Wounding stress induces the MKK3–MPK8 cascade, which activates MAPK, of which full activation of MPK8 also requires damage-induced Ca2+-dependent binding of calmodulin *CaM4* [31,32,33]. According to our results, the *CaM4* gene was upregulated in C119 at T2. The *CaM4* gene negatively regulates the accumulation of reactive oxygen species (ROS) by altering the early expression of the downstream gene RbohD to prevent ROS-induced plant damage. Similar findings on ROS and its effects on downstream genes have previously been reported in animal studies, but information related to plant species, particularly potato, is almost non-existent [34]. In C16, however, the expression level of *CaM4* was slightly increased until T12, and its degree of response to drought stress was delayed compared with C119 (Figure 6A,E). These results might explain why C16 is more sensitive to drought stress than C119. Therefore, upregulation of *CaM4* implies that C119 improves drought tolerance by preventing the excessive accumulation of ROS.

### 3.3. Phytohormone Signaling Pathways Are Involved in the Drought Stress Response in C119 and C16

Plant hormones are key signals that regulate plant growth, development, and stress tolerance [35,36]. Understanding the correlation between drought-mediated biochemical changes and plant hormone signaling pathways is important for improving the environmental adaption of plants through genetic engineering.

We found that the signaling pathways of six plant hormones, including auxin, JA, CK, SA, GA, and brassinolide, responded to the drought treatment, although the response patterns differed between the two genotypes. Auxin is transported into the cell through the *AUX1* vector and binds to the receptor TIR1, which then interacts with and ubiquitinates *AUX*/*IAA* [37]. The ubiquitination of *AUX*/*IAA* releases auxin response factors (ARFs) from the inhibitory control of *AUX*, thereby accelerating the transfer of auxin signal to promote cell expansion and plant growth; this is consistent with the results obtained in a previous study on Arabidopsis [37]. In the current study, the expression level of the gene encoding GH3 in C16 was slightly upregulated in C16 at T6 and T24, which likely promoted cell expansion and plant growth. At T24, the *AUX1* gene, which encodes *AUX*/*IAA*, was downregulated in C16; this presumably prevented *AUX* from activating ARF activity, consequently inhibiting the expansion and growth of plant cells. However, at T2 and T24, the expression level of *AUX*/*IAA* was upregulated in C119, leading to enhanced cell expansion and growth at the early (T2) and late (T24) stages of drought stress (Figure 7A,B). The regulatory effect of auxin on ARF activity has been observed in several previous studies [38,39]. However, what is highly noteworthy about our finding is that the expression levels of genes encoding the same protein varied between the two genotypes across various time points, indicating that the drought stress responses of the two genotypes differ at the molecular level. Auxin, a key regulator of root architecture and growth, is closely related to the drought resistance of plants, as indicated in this study. The signal transduction pathways differed considerably between C16 and C119 under drought stress. Hence, the key DEGs identified in this study could be used as candidate genes for the development of deep-rooting drought-resistant potato cultivars, which is worthy of functional verification and further mechanism-oriented research. 

Meesters et al. [40] proposed that JA could be converted to active JA-Ile in the JA signal transduction pathway by the JAR1-mediated addition of isoleucine. After a large amount of active JA-Ile accumulates, it binds to the JA receptor COI1, forming a complex, which promotes the degradation of the negative regulatory factor jasmonate ZIM-domain (*JAZ*) via ubiquitination [41,42]. In the current study, the expression level of *JAZ* was upregulated in C16 at T6 and T24, thereby activating the expression of downstream JA-responsive genes and regulating plant senescence and drought stress response. In C119, however, we did not find any significant changes in the expression levels of related genes (Figure 7A,C).

Marco et al. [43] reported that CK binds to the extracellular domain of the plasma-membrane-bound CRE1 receptor, inducing CRE1 autophosphorylation. The phosphate group of CRE1 is then transferred to the phosphate transporter AHP in the cytoplasm. The phosphorylated AHP enters the nucleus and transfers the phosphate group to the *B-ARRs*, and the phosphorylated *B-ARRs* then activate the expression of downstream genes, including *A-ARRs*, as indicated in previous studies [44,45]. Shi et al. [46] found that inhibiting the expression of *A-ARR* genes promotes cell division and negatively regulates freezing tolerance in Arabidopsis. Similarly, in our study, the *ARR* genes were upregulated at T24, which likely inhibited the activity of *B-ARRs*, thereby inhibiting cell division. In C16, because of the downregulation of *A-ARRs*, cell division would greatly increase. In C119, *B-ARRs* and *A-ARRs* were upregulated at T2, temporarily inhibiting cell division. With the increase in drought stress duration, the *B-ARR* and *A-ARR* genes were downregulated at T24, thereby promoting cell division (Figure 7A,D).

The SA signal transduction pathway and its effects on the *TGA* transcription factor have been well-documented previously. For example, Maheswari et al. [47] and Spoel et al. [48] showed that SA binds to NPR1 in the cytoplasm and dissociates the NPR1 oli-gomer into monomers, and then the monomeric form of NPR1 interacts with *TGA* after entering the nucleus. In C119, the *TGA* gene was downregulated at T2, which likely compromised plant stress resistance, and then was significantly upregulated at T12. Similarly, at T24, the expression levels of *TGA* and *PR-1* decreased, compromising plant stress resistance. However, in C16, we did not find any genetic changes in this pathway (Figure 7A,E). The role of SA in drought resistance has been extensively investigated in recent years, but its behavior in potato plants has rarely been reported. Since the drought stress response downstream of the SA pathway was dramatically different between the two genotypes, the key elements involved in this pathway should be investigated further in future studies.

In the GA signal transduction pathway, GA binds to the receptor GID1 in the nucleus, which then binds to the repressor protein DELLA, forming a stable complex. However, the binding to GID1 alters the spatial conformation of the DELLA protein, which induces the ubiquitination and subsequently degradation of DELLA by the 26S proteasome [49,50,51]. In C119, the expression level of *TF* was upregulated at T6, thereby promoting plant growth. However, in C16, the GA signaling genes showed no changes in expression levels (Figure 7A,F). This phenomenon may be related to the different drought tolerance mechanisms of the two genotypes.

In the brassinolide signal transduction pathway, BR binds to the extracellular domain of BRI1, leading to its phosphorylation. The phosphorylated BR1 then activates the kinase protein BSK, which then phosphorylates BSU1 phosphatase. The phosphorylated BR1 dephosphorylates BIN2, thereby activating key transcription factors BES1/BZR1 and the downstream gene *TCH4* and promoting the elongation and growth of cells [52,53]. At T2, the expression level of *TCH4* was upregulated in both C16 and C119, but its expression level changed more in C119 than in C16, indicating that BR synthesis is enhanced in C119 at the beginning of the drought stress, which confers a greater advantage to C119 over C16 in maintaining cell elongation and growth at the early stage of drought stress (Figure 7A,G).

### 3.4. Proposed Molecular Model of the Drought Stress Response in Potato

**Figure 9:**
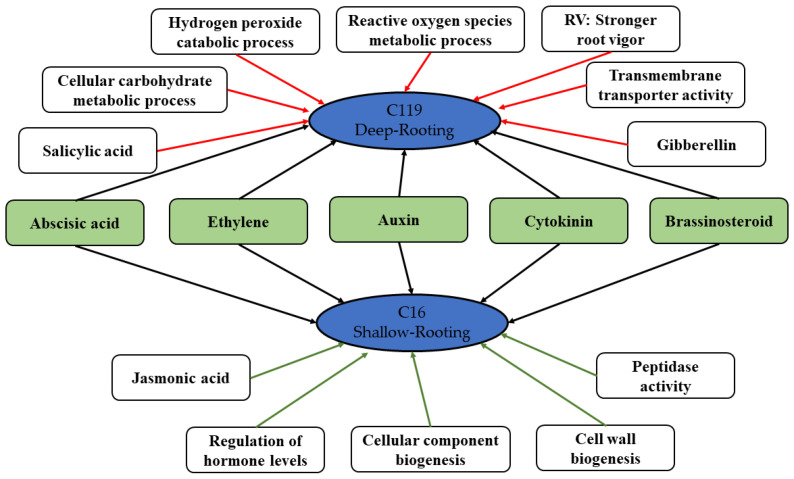
Molecular model of drought tolerance in the deep-rooting (C119) and shallow-rooting (C16) potato cultivars. Red and green lines represent the unique regulatory mechanisms in C119 and C16, respectively, and black lines represent the regulatory mechanism common to both C119 and C16.

We developed a drought tolerance model of potato based on the comparative analysis of transcriptomic and physiological data of two potato cultivars with varying degrees of tolerance to drought stress (Figure 9). Both potato cultivars, to some extent, shared a common mechanism of drought stress response, i.e., regulation of the signal transduction of hormones including ABA, ethylene, auxin, CK, and BR to resist the drought-stress-induced injury. However, C119 is more drought tolerant than C16, because of the following reasons: stronger root vigor; synergistic action of antioxidant enzymes, which eliminates harmful free radicals and alleviates oxidative stress; specific transduction of SA and GA signals; enhanced ROS metabolism metabolic process of reactive oxygen species; and greater ability to regulate the activity of transmembrane transporters and to reduce the drought-stress-induced damage to the cell membrane.

## 4. Materials and Methods

### 4.1. Plant Material 

Two potato genotypes, C16 (CIP 397077.16) and C119 (CIP 398098.119), were provided by the International Potato Research Center (Peru). Both genotypes showed the same growth cycle but different root structures and drought resistance levels (Table 1). The in vitro grown seedlings of C16 and C119 were provided by the Key Laboratory of Crop Genetic Improvement and Germplasm Innovation of Gansu Agricultural University.

### 4.2. Experiment Design

The stem segments of potato tube seedings were transferred to paper boats floating on normal liquid Murashige and Skoog (MS) medium. After 25 days of growth, the paper boats containing the tube seedlings were carefully taken out and placed in liquid MS medium containing 0 mM (control) or 150 mM mannitol (drought stress treatment). The seedlings were treated with mannitol for 0 h (T0), 2 h (T2), 6 h (T6), 12 h (T12), and 24 h (T24). Three biological replicates were conducted for each treatment. Subsequently, the seedlings roots were collected, immediately frozen in liquid nitrogen, and stored at −80 °C. Half of each stored sample was used for RNA extraction and subsequent transcriptome sequencing, while the other half was used for the determination of physiological and biochemical indices related to drought stress resistance.

### 4.3. Acquisition and Analysis of Transcriptome Data

A total of 30 root samples were collected (2 genotypes × 5 time points × 3 replicates) for transcriptome sequencing, and data were analyzed based on the potato reference genome sequence and its annotation files, retrieved from the Ensembl website (http://plants.ensembl.org/Solanum_tuberosum/Info/Index). The sequence reads were compared with the potato reference genome using Hisat2 (v2.0.5, http://ccb.jhu.edu/software/tophat/index.shtml (accessed on 20 June 2022)) [54,55]. Then, SAM files were converted to BAM files using Samtools (v0.1.19, http://samtools.sourceforge.net (accessed on 20 June 2022)), and reordered [56,57]. Cufflinks (v2.0.2, http://cufflinks.cbcb.umd.edu/ (accessed on 20 June 2022)) [58,59] was then used to assemble the transcripts, estimate transcript abundance based on the sorted files, and detect differential expression and variable shears between samples. The high-throughput data were processed using the HTSeq (v2.0.2, https://htseq.readthedocs.io/en/master/ (accessed on 20 June 2022)) [60,61] package in Python, and the expression level of each gene was reported as the FPKM value (fragments per kilobase of transcript per million mapped reads), calculated based on the read length and the number of reads mapped on to a specific gene. In general, when the absolute value of FPKM of a gene is greater than 1, it is considered to be expressed. The differences in gene expression levels among samples were then analyzed using the DESeq2 (http://www.bioconductor.org/packages/release/bioc/html/DESeq2.html (accessed on 20 June 2022)) package of R software (v3.5.2) [62,63]. To better locate the core genes, the DEGs with |log2FC| > 1 and *p* < 0.05 were used for subsequent screening. The cluster profiler package, based on the R language, was used for GO and KEGG enrichment analyses [64,65]. The GO and KEGG categories with *p* < 0.05 were considered significantly enriched.

### 4.4. RNA-seq Data Validation 

Twelve randomly selected genes with different expression patterns, as determined by RNA-seq, were subjected to qPCR. Total RNA extracted from three biological replicates of each treatment (T0, T2, T6, T12, and T24) was used for this task. First-strand cDNA was synthesized using the PrimeScript RT kit (TAKARA BIO Inc., Shiga, Japan). Gene-specific primers for qPCR were designed using the Primer5 software (Table S1). *Actin I* was used as an internal reference gene [66]. The qPCR was carried out on Quant Studio 5 (Life Technologies Holdings Pte Ltd., Singapore) using SYBR Premix Ex Taq II (Tli RNaseH Plus; TAKARA BIO Inc., Shiga, Japan). Three replicates were conducted for each gene. The ggpubr package of R was used to verify the qPCR and RNA-seq data of all samples.

### 4.5. Functional Annotation and GO and KEGG Classification of Genes

All expressed genes were functionally annotated by BLASTX searches (with e-value cutoff of 1 × 10^−5^ in Blast2GO) against four databases, including the NCBI (https://www.ncbi.nlm.nih.gov/ (accessed on 20 June 2022)) nonredundant (nr) proteins, Clusters of Orthologous Groups of proteins (COG), KEGG (https://www.kegg.jp/ (accessed on 20 June 2022)), and Swiss-Prot (https://ngdc.cncb.ac.cn/databasecommons/database/id/5614 (accessed on 20 June 2022)) [67]. If a gene matched to multiple protein sequences, the one with the highest similarity score was considered as its optimal annotation.

Genes upregulated in C16 and C119 at each time point were subjected to GO enrichment analysis in WEGO (https://wego.genomics.org.cn/ (accessed on 20 June 2022)) [68]. A χ^2^ test was used to further evaluate the gene portion differences, based on the GO classification results of the two genotypes. For each KEGG pathway, the numbers of genes up- and downregulated in each genotype were compared to the reference value established by Fisher’s Exact Test. Both GO and KEGG enrichment analyses were used for analyzing all DEGs at different time points.

## 5. Conclusions

This study presents two new major findings: (1) the drought tolerance of potato is highly correlated with the sensitivity of transcriptional responses to drought stress; (2) four *MAPKs* participate in ABA, ethylene, H_2_O_2_ and wounding signal transduction pathways, and the signaling pathways of six plant hormones including auxin, JA, CK, SA, GA, and brassinolide are involved in controlling the drought stress resistance of potato. Among the plant hormone signaling pathways, the JA signaling pathway was highly involved in C16, and the ABA, ethylene, and SA signaling pathways were highly involved in C119. These genes that respond to different hormone signaling pathways play an important role in potato root response to drought stress Additionally, the major DEGs could be applied for the development of drought-tolerant germplasm in the future. These new findings greatly enhance our understanding of the genetic and molecular bases of drought tolerance in two unique potato genotypes and provide important clues for the molecular breeding of drought-tolerant varieties.

## Figures and Tables

**Table 1:** Summary of the plant material used in this study.

Variety	CIP Number	Maternal Parent	Paternal Parent	Total Root Length (cm)	No. of Branches	Root Area (cm^2^)	Root Volume (cm^3^)
**C16**	CIP397077.16	392025.7 (=LR93.221)	392820.1 (=C93.154)	83.92	75.23	11.35	107.33
**C119**	CIP398098.119	393371.58	392639.31	121.07	119.67	12.77	122.45

Note: Root system data were derived from test-tube seedlings grown for 25 days under normal conditions.

## Data Availability

The original contributions presented in the study are publicly available. This data can be found here: National Center for Biotechnology Information (NCBI) BioProject database under accession number PRJNA646050.

## Supplementary Materials

The following supporting information can be downloaded at: https://www.mdpi.com/article/10.3390/plants11152024/s1, Figure S1: Comparison between the RNA-seq and qPCR data of 12 randomly selected genes; Figure S2: Gene ontology (GO) analyses of DEGs identified in T2 vs. T0 (A), T6 vs. T2 (B), T12 vs. T6 (C), and T24 vs. T12 (D) comparisons; Table S1: Primers used for qRT-PCR analysis in this study.
