# Supplementary material for: Comparative Transcriptome Analysis of Deep-Rooting and Shallow-Rooting Potato (Solanum tuberosum L.) Genotypes under Drought Stress
Source: Plants (Basel). 2022 Aug 3;11(15):2024. doi: 10.3390/plants11152024 (PMC9370241; doi:10.3390/plants11152024)
Supplement: Supplementary file 1 [file plants-11-02024-s001.zip › Figure S1.pdf]

Figure S1. Comparison of the randomly selected twelve genes expression between RNA sequencing and qPCR.

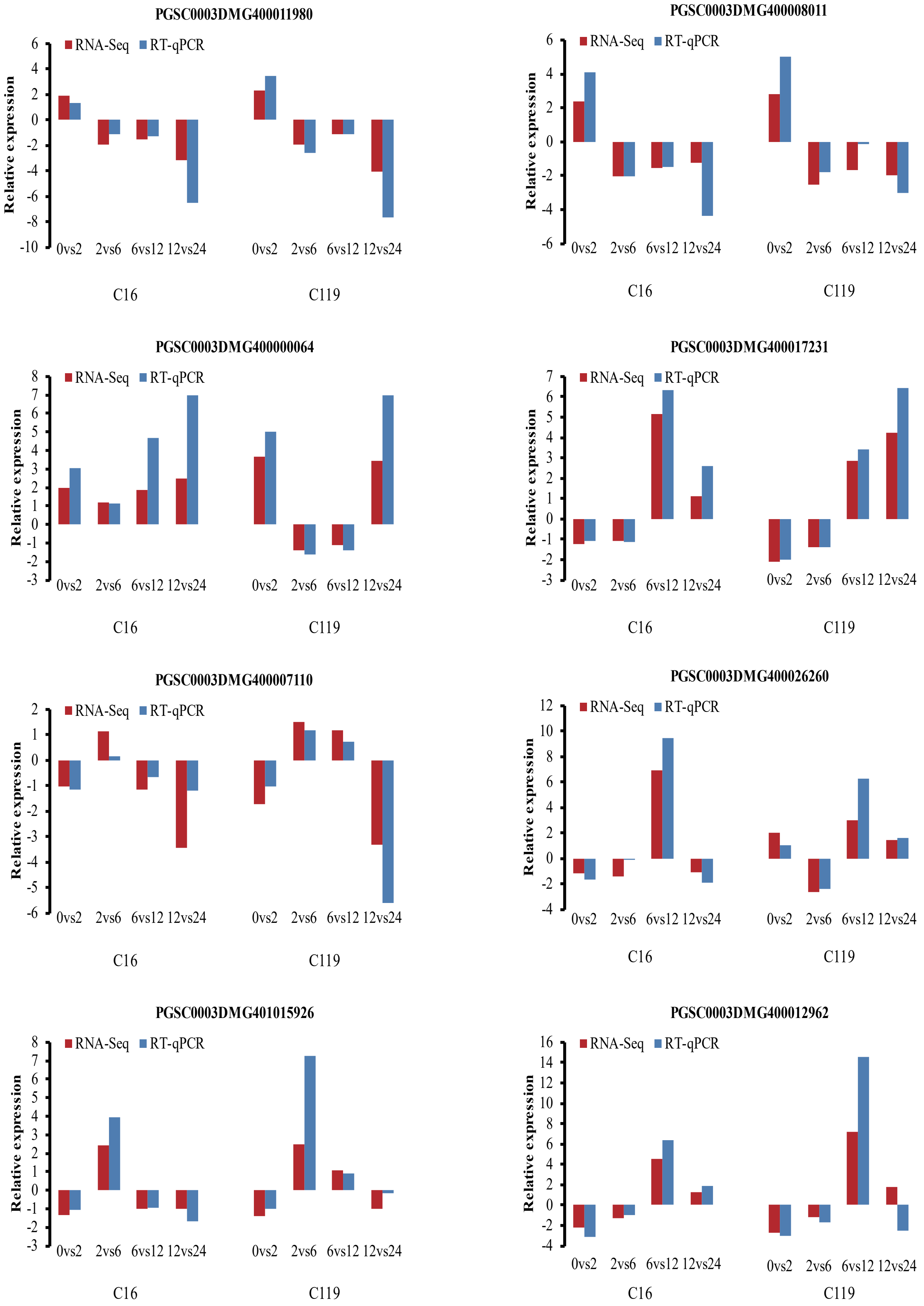

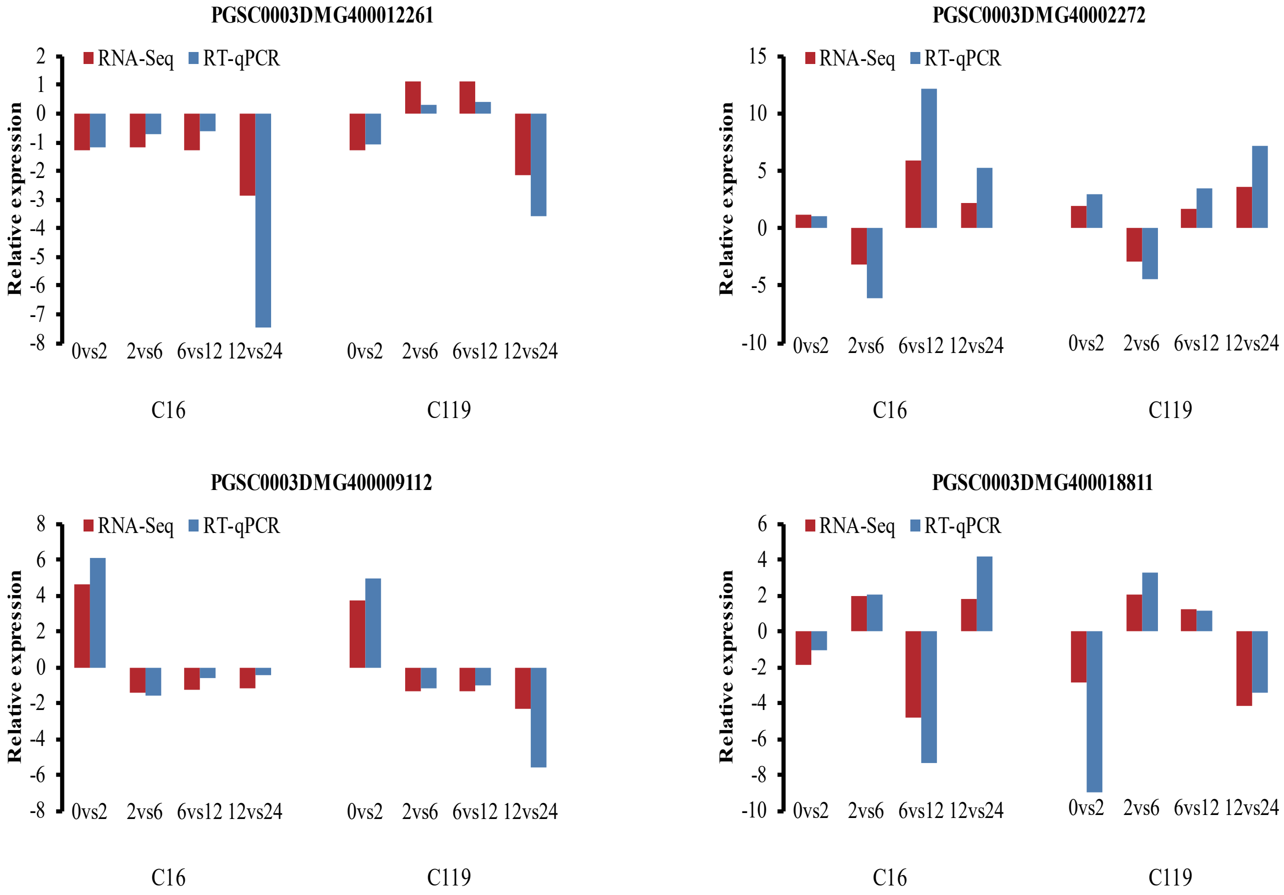
PGSC0003DMG400012261
RNA-Seq
RT-qPCR
Relative expression
2
1
0
-1
-2
-3
-4
-5
-6
-7
-8
0vs2
2vs6
6vs12
12vs24
C16
C119
PGSC0003DMG40002272
15
10
5
0
-5
-10
PGSC0003DMG400009112
8
6
4
2
0
-2
-4
-6
-8
PGSC0003DMG400018811
6
4
2
0
-2
-4
-6
-8
-10

Figure S1
