# Supplementary material for: Comparative Transcriptome Analysis of Deep-Rooting and Shallow-Rooting Potato (Solanum tuberosum L.) Genotypes under Drought Stress
Source: Plants (Basel). 2022 Aug 3;11(15):2024. doi: 10.3390/plants11152024 (PMC9370241; doi:10.3390/plants11152024)
Supplement: Supplementary file 1 [file plants-11-02024-s001.zip › Figure S2.pdf]

Figure S2. Gene ontology (GO) analyses for the DEGs of the two potato genotypes at T2 vs.T0 (A), T6 vs.T2 (B), T12 vs.T6 (C) and T24 vs.T12 (D). * indicates significant correlation at p< 0.05 level, while ** indicates a highly significant correlation at p< 0.01 level.

A

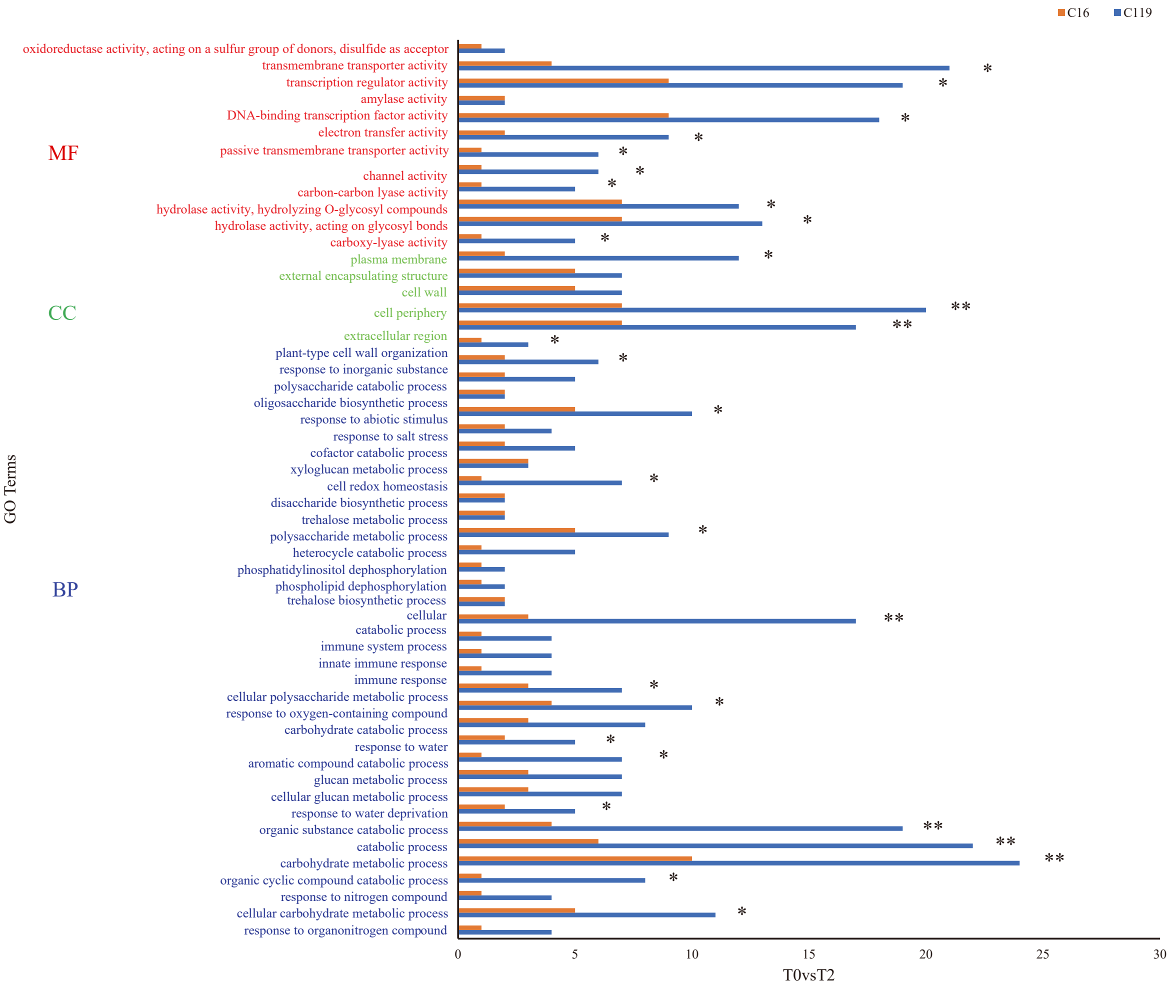

B

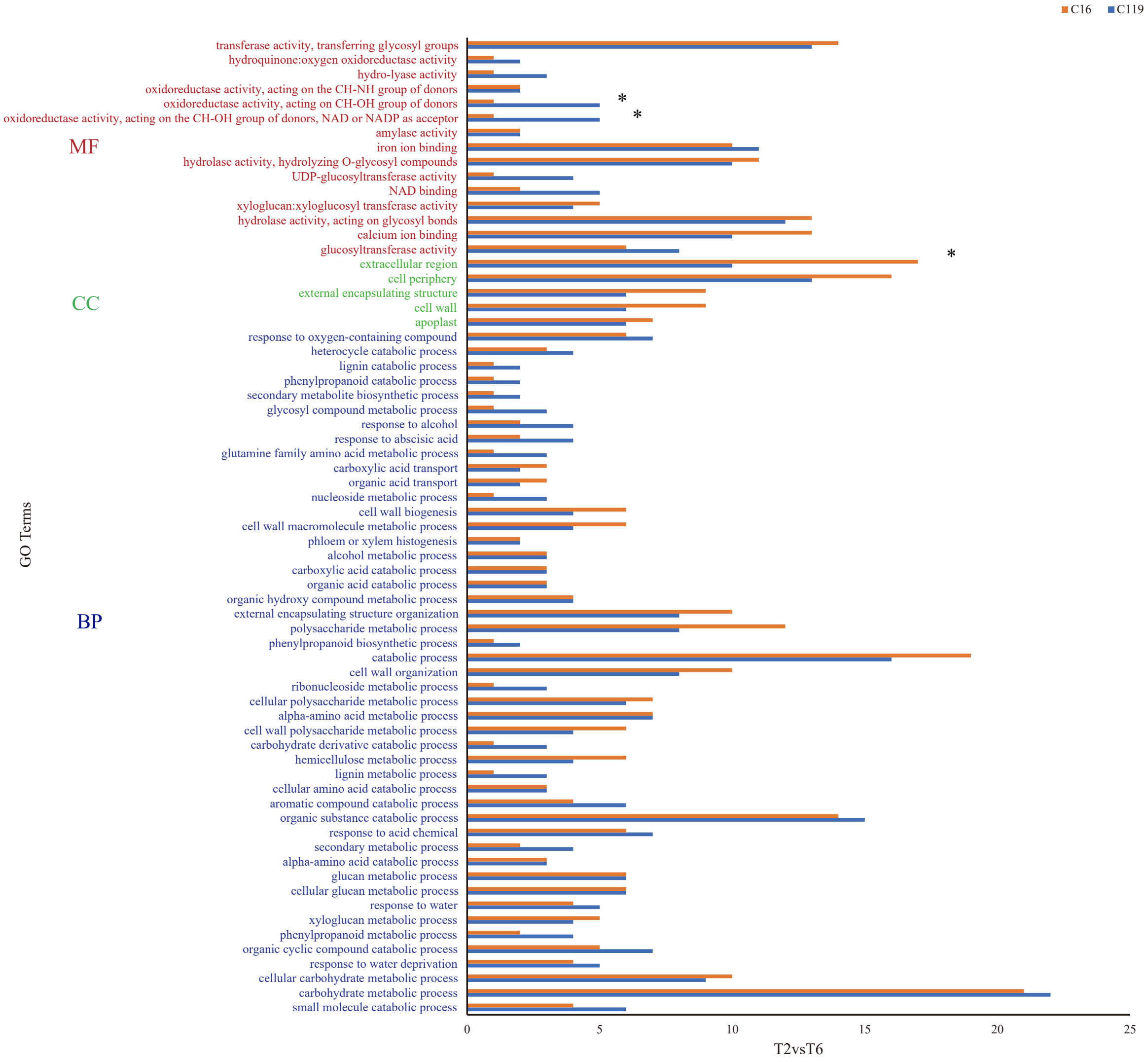

C

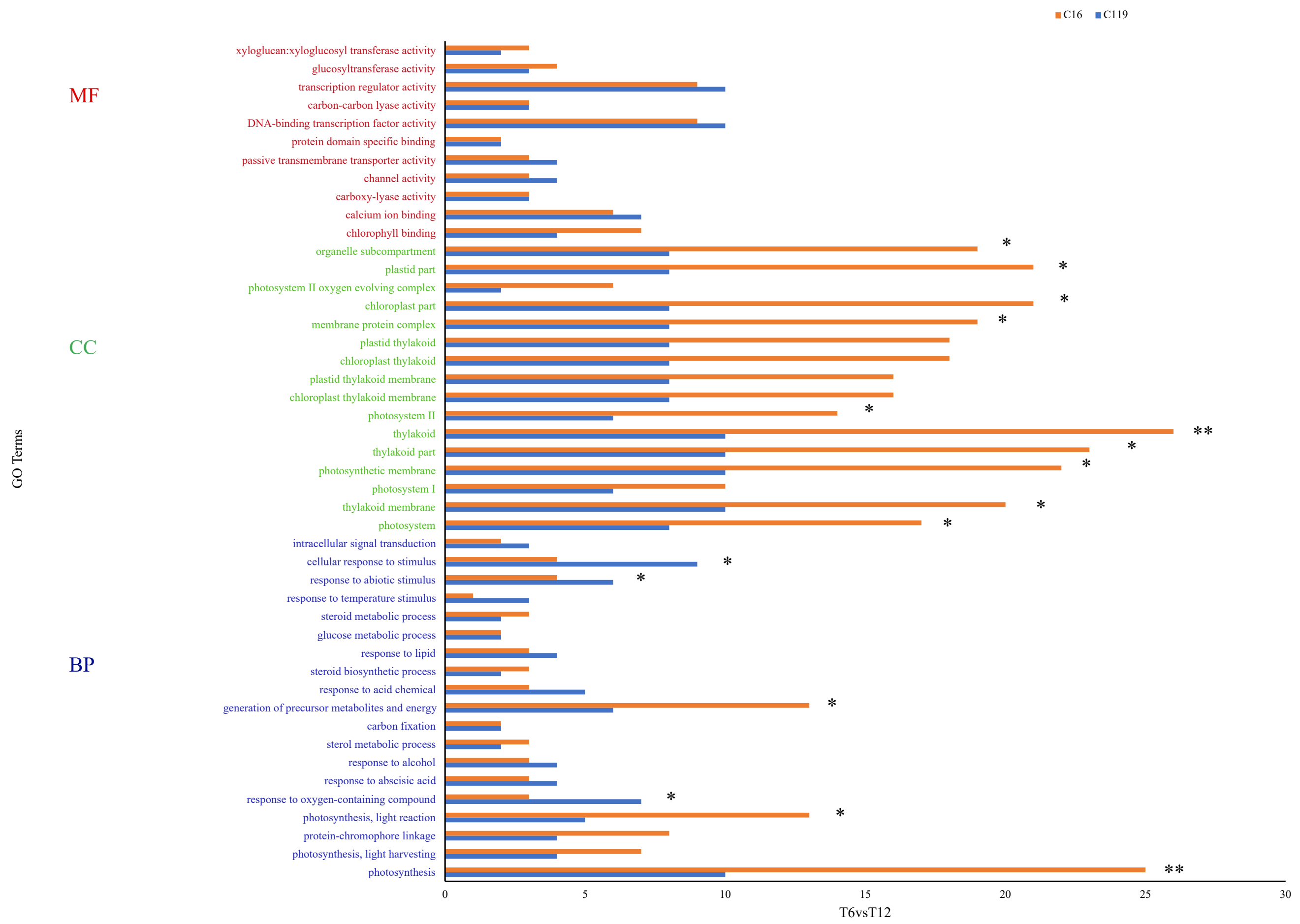

C16
C119
MF
CC
BP
GO Terms
xyloglucan:xyloglucosyl transferase activity
glucosyltransferase activity
transcription regulator activity
carbon-carbon lyase activity
DNA-binding transcription factor activity
protein domain specific binding
passive transmembrane transporter activity
channel activity
carboxy-lyase activity
calcium ion binding
chlorophyll binding
organelle subcompartment
plastid part
photosystem II oxygen evolving complex
chloroplast part
membrane protein complex
plastid thylakoid
chloroplast thylakoid
plastid thylakoid membrane
chloroplast thylakoid membrane
photosystem II
thylakoid
thylakoid part
photosynthetic membrane
photosystem I
thylakoid membrane
photosystem
intracellular signal transduction
cellular response to stimulus
response to abiotic stimulus
response to temperature stimulus
steroid metabolic process
glucose metabolic process
response to lipid
steroid biosynthetic process
response to acid chemical
generation of precursor metabolites and energy
carbon fixation
sterol metabolic process
response to alcohol
response to abscisic acid
response to oxygen-containing compound
photosynthesis, light reaction
protein-chromophore linkage
photosynthesis, light harvesting
photosynthesis
0
5
10
15
20
25
30
T6vsT12

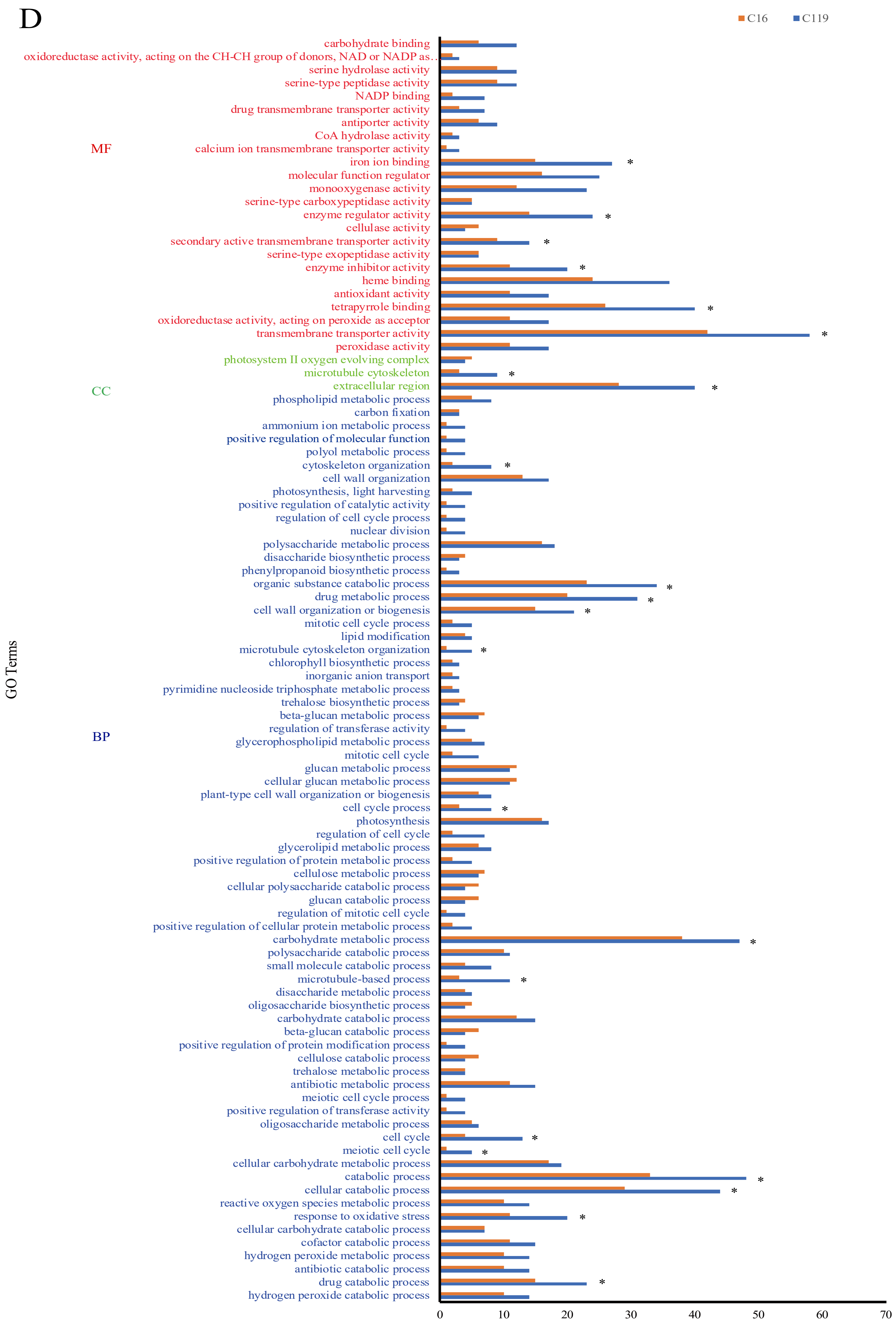
D
C16
C119
MF
carbohydrate binding
oxidoreductase activity, acting on the CH-CH group of donors, NAD or NADP as…
serine hydrolase activity
serine-type peptidase activity
NADP binding
drug transmembrane transporter activity
antiporter activity
CoA hydrolase activity
calcium ion transmembrane transporter activity
iron ion binding
molecular function regulator
monooxygenase activity
serine-type carboxypeptidase activity
enzyme regulator activity
cellulase activity
secondary active transmembrane transporter activity
serine-type exopeptidase activity
enzyme inhibitor activity
heme binding
antioxidant activity
tetrapyrrole binding
oxidoreductase activity, acting on peroxide as acceptor
transmembrane transporter activity
peroxidase activity
CC
photosystem II oxygen evolving complex
microtubule cytoskeleton
extracellular region
BP
phospholipid metabolic process
carbon fixation
ammonium ion metabolic process
positive regulation of molecular function
polyol metabolic process
cytoskeleton organization
cell wall organization
photosynthesis, light harvesting
positive regulation of catalytic activity
regulation of cell cycle process
nuclear division
polysaccharide metabolic process
disaccharide biosynthetic process
phenylpropanoid biosynthetic process
organic substance catabolic process
drug metabolic process
cell wall organization or biogenesis
mitotic cell cycle process
lipid modification
microtubule cytoskeleton organization
chlorophyll biosynthetic process
inorganic anion transport
pyrimidine nucleoside triphosphate metabolic process
trehalose biosynthetic process
beta-glucan metabolic process
regulation of transferase activity
glycerophospholipid metabolic process
mitotic cell cycle
glucan metabolic process
cellular glucan metabolic process
plant-type cell wall organization or biogenesis
cell cycle process
photosynthesis
regulation of cell cycle
glycerolipid metabolic process
positive regulation of protein metabolic process
cellulose metabolic process
cellular polysaccharide catabolic process
glucan catabolic process
regulation of mitotic cell cycle
positive regulation of cellular protein metabolic process
carbohydrate metabolic process
polysaccharide catabolic process
small molecule catabolic process
microtubule-based process
disaccharide metabolic process
oligosaccharide biosynthetic process
carbohydrate catabolic process
beta-glucan catabolic process
positive regulation of protein modification process
cellulose catabolic process
trehalose metabolic process
antibiotic metabolic process
meiotic cell cycle process
positive regulation of transferase activity
oligosaccharide metabolic process
cell cycle
meiotic cell cycle
cellular carbohydrate metabolic process
catabolic process
cellular catabolic process
reactive oxygen species metabolic process
response to oxidative stress
cellular carbohydrate catabolic process
cofactor catabolic process
hydrogen peroxide metabolic process
antibiotic catabolic process
drug catabolic process
hydrogen peroxide catabolic process
GO Terms
0
10
20
30
40
50
60
70
T12vsT24

Figure S2
